# Publisher Correction: Schools of skyrmions with electrically tunable elastic interactions

**DOI:** 10.1038/s41467-020-14419-5

**Published:** 2020-01-29

**Authors:** Hayley R. O. Sohn, Changda D. Liu, Ivan I. Smalyukh

**Affiliations:** 1 Department of Physics and Materials Science and Engineering Program, University of Colorado, Boulder, CO 80309 USA; 2 Department of Electrical, Computer, and Energy Engineering and Soft Materials Research Center, University of Colorado, Boulder, CO 80309 USA; 3 Renewable and Sustainable Energy Institute, National Renewable Energy Laboratory and University of Colorado, Boulder, CO 80309 USA

**Keywords:** Topological defects, Liquid crystals, Self-assembly, Topological defects

Correction to: *Nature Communications* 10.1038/s41467-019-12723-3, published online 18 October 2019.

The original version of this Article contained an error in the equation in the section ‘Emergence of polar order and coherent motions’ under ‘Results’. The equation incorrectly reads: $S = |\mathop {\sum }\limits_i^N {\mathbf{v}}_i|/\left( {N_{V_s}} \right)$.

The correct form of this equation is: $S = |\mathop {\sum }\limits_i^N {\mathbf{v}}_i|{\mathrm{/}}\left( {N\,{\mathbf{v}}_s} \right)$.

The original version of this Article contained an error in Fig. 5d, in which the y-axis label incorrectly reads ‘$g(r_{cc})$’ rather than the correct ‘$\rho \left( {r_{cc}} \right)$.’

These errors have been corrected in both the PDF and HTML versions of the Article.

