# Supplementary material for: Schools of skyrmions with electrically tunable elastic interactions
Source: Nat Commun. 2019 Oct 18;10:4744. doi: 10.1038/s41467-019-12723-3 (PMC6802192; doi:10.1038/s41467-019-12723-3)
Supplement: Supplementary file 1 — Supplementary Information [file 41467_2019_12723_MOESM1_ESM.pdf]

Supporting Information

Schools of skyrmions with electrically tunable elastic interactions

Hayley R. O. Sohn[1], Changda D. Liu[1], and Ivan I. Smalyukh[1,2,3*]

[1]*Department of Physics and Materials Science and Engineering Program, University of Colorado, Boulder, CO 80309, USA*

[2]*Department of Electrical, Computer, and Energy Engineering and Soft Materials Research Center, University of Colorado, Boulder, CO 80309, USA*

[3]*Renewable and Sustainable Energy Institute, National Renewable Energy Laboratory and University of Colorado, Boulder, CO 80309, USA*

* *Correspondence to: ivan.smalyukh@colorado.edu*

Supplementary Figures

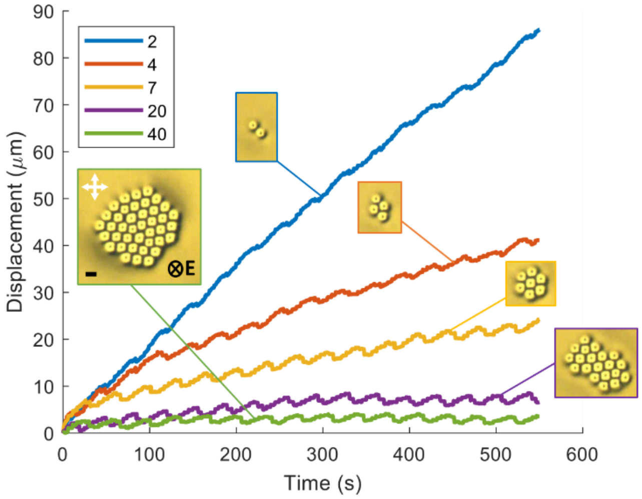

Supplementary Figure 1 | Skyrmion displacement versus time for different clusters. Displacement analysis of skyrmion clusters versus time at U=3.75 V at 2Hz oscillation frequency and at carrier frequency of 1kHz. The legend indicates the number of skyrmions in each cluster. Crossed polarizer orientations for all insets are marked with double arrows and the direction of **E** is shown. The scale bar is 10 μm. The LC is MLC-6609 doped with ZLI-811.

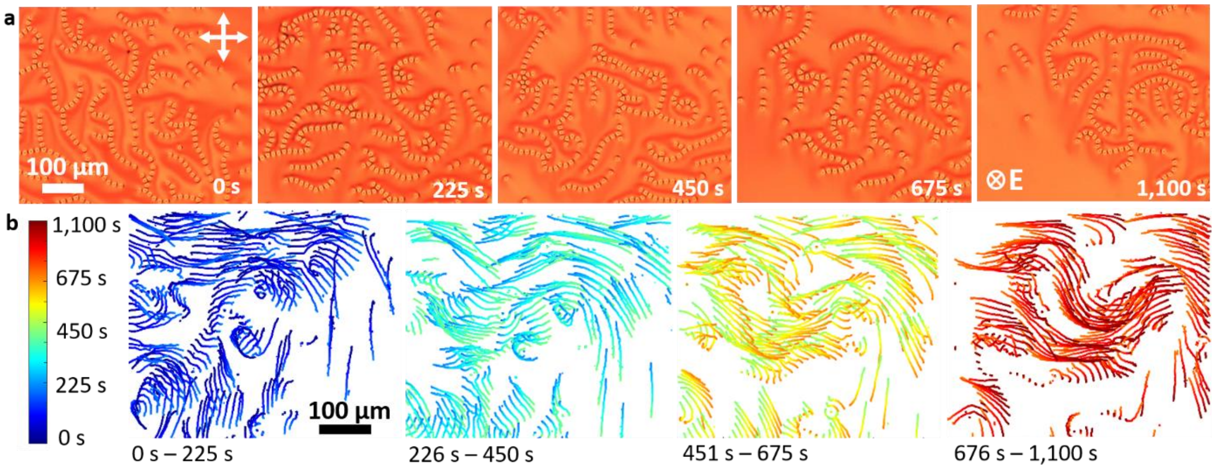

Supplementary Figure 2 | Details of the chain-like swirling motion of skyrmions. **a**, Temporal evolution of skyrmion chains at packing fraction ~0.005 and U=4.2V oscillated at f=2 Hz. **b**, Time-colored trajectories representing skyrmion motions between the frames displayed in (**a**), with the left-side inset showing the time-color scheme for corresponding time periods marked beneath the plots. White double arrows denote crossed polarizers for all micrographs in this figure. The direction of **E** marked in the last frame of (**a**) corresponds to all figure parts. The elapsed time is marked on the frames. The scale bars are 100 μm. The chiral LC is MLC-6609 doped with QL-76.
