# Supplementary material for: Schools of skyrmions with electrically tunable elastic interactions
Source: Nat Commun. 2019 Oct 18;10:4744. doi: 10.1038/s41467-019-12723-3 (PMC6802192; doi:10.1038/s41467-019-12723-3)
Supplement: Supplementary file 2 — Description of Additional Supplementary Files [file 41467_2019_12723_MOESM2_ESM.pdf]

Description of Additional Supplementary Files

File Name: Supplementary Movie 1
Description: Collective motion of dense clusters of skyrmions moving and rearranging together to avoid randomly-pinned obstacles (stationary skyrmions, marked by red dots). The motion is powered at U=3V and f=60 Hz. The video is played at 20x speed and the actual elapsed time is 405 s. White double arrows mark the crossed polarizer orientations. The chiral LC mixture is the nematic host ZLI-2806 doped with CB-15.

File Name: Supplementary Movie 2
Description: Skyrmion chain motion race between a trio-chain, a pair, and an individual skyrmion. The motion is powered by U=4.5 V voltage with oscillation at 2 or 8 Hz, as noted in top left of video frames, with carrier frequency of 1 kHz. The video is sped up 50 times. The net elapsed time is 29 minutes and 46 s. Crossed polarizer orientations are marked with white double arrows. The chiral LC mixture is the nematic MLC-6609 doped with the chiral additive ZLI-811.

File Name: Supplementary Movie 3
Description: Kinetic out-of-equilibrium self-assembly and re-assembly of skyrmion superstructures. The motion is powered at a U=4.5 V voltage, with oscillation at 2 or 8 Hz, as noted in top left of video frames; the carrier frequency is 1 kHz. The video is sped up approximately 100 times and the total elapsed time is 1 hour, 10 minutes and 18 s. White double arrows denote polarizer orientations. The LC is the nematic host MLC-6609 doped with the chiral additive ZLI-811.

File Name: Supplementary Movie 4
Description: Temporal evolution of self-assembled meandering chain motion in a swirling pattern shown in Fig.2d-f and Supplementary Figure 2. The motion is powered with U=4.2V at f=2 Hz oscillation, with 1 kHz carrier frequency. The video is sped up 50 times the actual elapsed time is 17 minutes. White double arrows represent the crossed polarizer orientations. The chiral LC is MLC-6609 doped with the chiral additive QL-76.

File Name: Supplementary Movie 5
Description: Large-scale collective motion of individually dispersed skyrmions within a school, powered at U=3.75 V and f=50 Hz. The video clip is sped up 3 times. The elapsed time is 65 s. Polarizer and analyzer orientation are marked with white and yellow double arrows. The chiral mixture is the nematic host ZLI-2806 doped with the chiral additive CB-15.

File Name: Supplementary Movie 6
Description: Large-scale collective motion of dynamically self-assembled skyrmion clusters powered with U=3.5 V at f=50 Hz. The video is sped up 10 times. The elapsed time is 234 s. Polarizer and analyzer orientations are marked with white and yellow double arrows. The chiral mixture is the nematic host ZLI-2806 doped with the chiral additive CB-15.

File Name: Supplementary Movie 7
Description: Large-scale collective motion of self-assembled chains of skyrmions electrically powered at U=4.0 V and f=50 Hz. The video is sped up 3 times. The actual elapsed time is 51 s. Polarizer and analyzer orientations are marked with white and yellow double arrows. The chiral mixture is the nematic host ZLI-2806 doped with the chiral additive CB-15.
